# Triangular Relationship between p53, Autophagy, and Chemotherapy Resistance

**DOI:** 10.3390/ijms21238991

**Published:** 2020-11-26

**Authors:** Jingwen Xu, Nipa H. Patel, David A. Gewirtz

**Affiliations:** 1 School of Pharmacy, Guangdong Pharmaceutical University, Guangzhou 510006, China; jingwen_xu@gdpu.edu.cn; 2 Massey Cancer Center, Goodwin Research Laboratories, Virginia Commonwealth University, Richmond, VA 23298, USA; patelnh3@vcu.edu; 3 Department of Pharmacology and Toxicology and Medicine, Virginia Commonwealth University, Richmond, VA 23298, USA

**Keywords:** p53, autophagy, chemoresistance

## Abstract

Chemotherapy and radiation often induce a number of cellular responses, such as apoptosis, autophagy, and senescence. One of the major regulators of these processes is p53, an essential tumor suppressor that is often mutated or lost in many cancer types and implicated in early tumorigenesis. Gain of function (GOF) p53 mutations have been implicated in increased susceptibility to drug resistance, by compromising wildtype anti-tumor functions of p53 or modulating key p53 processes that confer chemotherapy resistance, such as autophagy. Autophagy, a cellular survival mechanism, is initially induced in response to chemotherapy and radiotherapy, and its cytoprotective nature became the spearhead of a number of clinical trials aimed to sensitize patients to chemotherapy. However, increased pre-clinical studies have exemplified the multifunctional role of autophagy. Additionally, compartmental localization of p53 can modulate induction or inhibition of autophagy and may play a role in autophagic function. The duality in p53 function and its effects on autophagic function are generally not considered in clinical trial design or clinical therapeutics; however, ample pre-clinical studies suggest they play a role in tumor responses to therapy and drug resistance. Further inquiry into the interconnection between autophagy and p53, and its effects on chemotherapeutic responses may provide beneficial insights on multidrug resistance and novel treatment regimens for chemosensitization.

## 1. Introduction

Although the treatment of cancer has seen significant advances in recent years, chemotherapeutic drugs continue to represent a primary component of most current cancer therapies. However, drug resistance, and often multidrug resistance (MDR), are primary reasons for the failure of clinical chemotherapy [1]. 

Drug resistance can be intrinsic to the tumors or be acquired during treatment. Long-term sublethal drug exposure of tumor cells is one basis for the development of acquired drug resistance. Drug resistance often involves multiple mechanisms. For instance, physiological barriers such as a dense fibroblast envelope in the tumor tissue and the absence of lymphatic drainage can limit drug access to the tumor [2]. This is particularly relevant to efforts to treat pancreatic cancer [3,4]. The generation of acidic lysosomes in drug-resistant tumor cells forming ion traps that can bind weakly basic anticancer drugs (such as adriamycin hydrochloride and irinotecan hydrochloride) may contribute to reduced drug efficacy [5]. In addition, activation of multidrug-resistant proteins, inhibition of cell death pathways through imbalance in pro-apoptotic and anti-apoptotic proteins, changes in drug metabolism, epigenetic changes, or changes in drug targets may all lead to chemotherapy resistance.

In recent years, extensive evidence has accumulated for senescence as a primary response to cancer therapeutics. Therapy-induced senescence (TIS) provides an additional layer of complexity to predicting tumor cell fate after therapy in that recovery from senescence provides a potential pathway from drug and radiation lethality. Since subpopulations of tumor cells can enter into a transient senescent-mediated growth arrested state and subsequently regain proliferative capacity, senescence may represent one element of tumor dormancy state, which eventually facilitates disease recurrence, representing a mechanism of delayed drug resistance [6]. 

The primary intent of cancer treatment strategies is obviously to promote tumor cell death; therefore, factors affecting cell death and the underlying mechanisms are central issues in determining therapeutic efficacy. As the most well-studied tumor suppressor gene, p53 plays a “gate keeper” role in tumorigenesis [7,8]. Unfortunately, the p53 pathway is often inactivated or functions in an antagonistic manner towards drug effectiveness [9,10]. In fact, p53 was initially identified as an oncogene due to the inadvertent use of mutated p53 sequences from tumors [11]. The mutated form of p53 protein is often expressed in cancer, promoting cell transformation, metastasis and drug resistance, in part by inhibiting wildtype p53 (wtp53) [12]. Cancer genome sequencing has shown that 42% of the 12 tumor types studied carried the mutant *TP53* gene [9,13]. 

In tumor cells exposed to chemotherapy and radiotherapy, one of the first responses is autophagy, a cellular process that removes damaged proteins and organelles as well as generating energy and metabolic intermediates. Autophagy can promote or attenuate tumor resistance, depending on whether it is cytoprotective or cytotoxic in nature [14,15]. There is extensive evidence that p53 can modulate autophagy. Interestingly, p53 can play dual roles, where nuclear p53 induces autophagy through transcriptional effects, whereas cytoplasmic p53 acts as a master repressor of autophagy [16,17]. Hence, it is not surprising that tumors differing in p53 status may not have identical influence on autophagy function. This review attempts to provide a comprehensive summary of the effects of p53 status on the functional form of autophagy, which in turn modulates drug sensitivity and resistance. 

### 1.1. p53 and Drug Resistance

The p53 tumor suppressor protein, a transcription factor that can respond to various forms of exogenous stress and inhibit cell division or survival, is often considered to be the key fail-safe mechanism of cell anti-cancer defenses [18,19]. Consequently, in order to enhance their survival and/or maintain growth, cancer cells use a variety of strategies to disarm p53. The most direct and effective way to inactivate p53 is to mutate the p53-encoding gene *TP53* [20]. Since the frequent mutation of *TP53* in human cancers was described 30 years ago, the mutation patterns of *TP53* in cancers and the role of p53 in cancer etiology have been gradually clarified [21,22,23]. Mutations in p53 are the most common genetic lesion in cancers, and correspond with cancer development, progression, metastasis, and resistance to chemotherapy or radiotherapy. Most p53 mutations occur in the central DNA-binding domain, resulting in the loss of wildtype function (so-called loss of function, LOF) or have a dominant-negative effect on the wildtype alleles. Some mutations (such as R248Q, R273H, R175H, and R249S) have shown “gain of function” (GOF), which can further promote cancer malignancy and chemoresistance [10]. However, there are several DNA binding domain mutants, such as G245S and R246S variants, which do not exhibit any GOF properties [24,25]. The reason why only some p53 mutants express GOF properties are still unclear, but we might anticipate that drugs that directly target mutant p53 for degradation might be useful in improving the therapeutic responses. 

The multidrug resistance gene 1 (*MDR1*), also known as *ABCB1*, is often found to be over-expressed in cancer, encoding an ATP-dependent efflux pump, which is responsible for inducing broad-spectrum chemical resistance. After Chin et al. demonstrated transcriptional dependence of the MDR1 gene promoter on p53, the clinical correlations between therapeutic resistance and p53 mutations gained more prominence and attention [26,27]. It has also been shown that p53 mutants are able to actuate various survival signaling cascades, such as the NF-κB, PDGFRβ, mevalonate, proteasomal, or integrin pathways [28,29,30,31], and activate an independent set of target genes in cooperation with other transcription factors or cofactors (such as Pin1 [32] and PML [33] proteins), thereby promoting tumor cell survival and/or proliferation. 

The mutant form of p53 can confer resistance to apoptosis, thereby reducing tumor cell susceptibility to cell death [27,34]. Mutant p53 not only interferes with the transcriptional activity of wtp53 in the nucleus, but also abolishes the interaction between wtp53 and BCL-2 family proteins in the cytoplasm. For example, p53 dysfunction led to decreased apoptosis induction by BCL-2 antagonists ABT-737 in chronic lymphocytic leukemia cells [35]. The p53 mutation of GOF also mediates resistance to apoptosis for many commonly used chemotherapeutic agents. For instance, cross-resistance between doxorubicin and paclitaxel was induced by introduction of the R248Q p53 mutant into hepatocellular carcinoma p53-null Hep3B cells [36]. Another example is where knockdown of the R273H p53 mutant in human squamous cell carcinoma increased procaspase-3 levels and sensitized these cells to doxorubicin and methotrexate-induced apoptosis [37]. Due to the LOF or GOF of abnormal *TP53* in tumors, reintroducing p53 through a virus encoding wtp53 or converting mutant p53 to wildtype function may be a potential therapeutic strategy for increasing the susceptibility of tumor cells to apoptosis [38]. However, regardless of the attempts to restore p53 in tumors lacking p53 [39], with p53 missense mutations [40] or in tumors driven by oncogenes [41,42], it is still difficult to predict the nature of the p53-mediated response that will be evoked, whether it is conventional growth arrest, senescence, and/or apoptosis. It seems the most effective approach might be to combine the reintroduction of p53 function with conventional chemotherapy drugs to promote tumor cell apoptosis. Taken together, in chemotherapy, mutant p53 represents a key factor in cancer cell resistance to treatment. 

### 1.2. Autophagy and p53 in Cancer Treatment 

Autophagy, a process of self-degradation, represents a critical physiological catabolic mechanism of eukaryotic cells. Autophagy is necessary for cells to respond to nutrient starvation and other types of stressful conditions, such as hypoxia [43]. Consequently, it is not surprising that autophagy can often be detected in tumor cells exposed to chemotherapy or radiation [44]. In response to chemotherapy, autophagy may exhibit several functional forms, including a cytoprotective form, a cytotoxic form that either directly or indirectly promotes tumor cell death, and what we have termed a nonprotective form, which does not appear to directly influence cell proliferation or apoptosis [15]. However, it is still unclear why autophagy, a life process that maintains cell homeostasis via elimination of oncogenic protein substrates, toxic unfolded proteins, and damaged organelles, exhibits these inconsistent effects. Nevertheless, because of the various roles played by autophagy in cancer treatment, autophagy offers a high degree of potential for future therapy. 

Indeed, at present, the majority of clinical studies that involve autophagy are in the field of cancer therapy. Currently, the mainstream clinical trials involve the combined use of chemotherapeutic drugs and chloroquine (CQ) or hydroxychloroquine (HCQ), based on the largely unproven premise that all therapies promote the cytoprotective form of autophagy in patient malignancies [45]. Published clinical trials have demonstrated the safety of CQ or HCQ; moreover, increased radiosensitivity and prolonged patient survival has been evident primarily in the treatment of glioblastoma [46,47,48]. Generally, however, the results of these clinical trials of CQ or HCQ combined with chemotherapy have been largely inconsistent, indicating the challenge of extrapolating to the clinical situation from in vitro and in vivo preclinical studies. In part, the inconsistency evident in clinical trials may be attributed to the inability to achieve sufficient HCQ or CQ plasma levels required to inhibit autophagy [49]. In fact, significantly higher doses would likely be required to effectively achieve autophagy inhibition in patient tumors, which would result in severe toxic side effects [49]. Furthermore, the optimal time frames for administration of autophagy inhibitors to maximize sensitization to chemotherapy or radiation therapy have also generally not been considered in the design of these clinical trials. Studies have shown that for normal non-cancerous cells, autophagy is necessary for the maintenance of cellular homeostasis, while mice lacking ATG5 or ATG7 have spontaneous defects [50,51,52]. Shingu et al. found that inhibition of autophagy at a late stage enhanced imatinib-induced cytotoxicity in human malignant glioma cells, but attenuated the imatinib-induced cytotoxicity at an early stage [53]. These studies suggest that if autophagy inhibitors are applied in the early stage of tumor formation, there is a risk of promoting tumorigenesis. 

Another issue that should not be ignored is the relationship between autophagy and the immune system. It has been demonstrated that autophagy has a surveillance effect on immunity, and a reduction of autophagy is related to the infiltration of regulatory T cells, which inhibit the immune system and reduce effective immune surveillance, thereby stimulating a tumor-promoting microenvironment [54]. Autophagy also plays a key role in processing of DAMPs, cytokine, and chemokine release for immune infiltration and recruitment, as well as processing of tumor-antigens for MHC presentation [55]. In addition, while autophagy deficiency has been shown to increase chemo- and radiosensitivity in immune-deficient mice [56,57], inhibition of autophagy in immune-competent mice has been reported to result in a failure of chemotherapy [58,59]; oddly, despite having been published almost 10 years ago, the latter study has not been independently verified by additional reports. This background further suggests that in the initial stage of tumor formation, when the immune system plays a critical role, inhibiting autophagy may accelerate the occurrence and progression of tumors. For now, the optimal staging for the use of autophagy inhibitors is in advanced tumors, which is also an ideal stage for the clinical trials that have been completed. 

Although many factors are likely to influence autophagy, the involvement of p53 cannot be ignored. As mentioned above, p53 plays a regulatory role in tumor cell proliferation, cell cycle regulation, apoptosis, senescence, and autophagy. This regulatory role is related to its subcellular localization. Studies have shown that p53 located in the nucleus promotes autophagy under stress, while cytosolic p53 inhibits autophagy in unstressed cells [16,17]. In the nucleus, p53 induces autophagy by regulating the mTOR pathway in a transcription-dependent manner, as well as transcriptional regulation of key autophagy-related genes (ATGs) [39,60]. Some p53 targeted genes, including PTEN, TSC2, and AMPKβ, have been reported to negatively regulate mTOR [61], thus promoting autophagy initiation. In addition, p53 can also be associated with autophagy through the p14ARF (p19ARF in mouse, and hereafter referred to as ARF)-signaling pathway [62]. During tumorigenesis, the activation of oncogenes upregulates the transcription of ARF, which in turn binds to and inhibits the expression of MDM2, thereby stabilizing p53 [19]. ARF positively regulates autophagy by disrupting the Bcl-xl/Beclin 1 complex, releasing Beclin-1 to induce autophagy [62]. 

Subcellular localization of p53 can also mediate functional responses between apoptosis and autophagy. In the cytosol, p53 can localize to the mitochondria where it can interact with antiapoptotic *BCL* family proteins, allowing oligomerization of proapoptotic factors, such as BAX and BAK, thereby promoting mitochondrial outer membrane permeabilization (MOMP) and driving activation of intrinsic apoptotic cell death pathway [63,64]. Studies by Tomita et al. demonstrated several breast cancer cell lines expressing various p53 mutants failed to form complexes with Bcl2 in MDA-MB-231 (p53 R280K), MDA-MB-468 (p53 R273H), T47D (p53 L194F), and SKBr3 (p53 R175H) cells when compared to ML-1 (wtp53) cells [65]. Furthermore, these p53 mutant breast cancer cell lines exhibited impaired mitochondrial permeabilization when compared to wtp53 cells. 

High mobility group box 1 (HMGB1), a conserved nuclear protein, acts as a chromatin-binding factor, binds to DNA, and promotes access to transcriptional protein complexes [66]. Similar to p53, the biological function of HMGB1 is related to its subcellular location [67]. Besides its nuclear effect, HMGB1 plays an important role in the processes of inflammation, cell differentiation, cell migration, wound healing, and tumor progression [68,69,70]. In addition to being a Beclin1-binding protein [71], cytoplasmic HMGB1 maintains the activation of the Beclin1-PtdIns3KC3 complex during the upregulation of autophagy [72]. HMGB1 also forms a complex with p53 and affects the cytoplasmic localization of reciprocal binding partners, thereby regulating the subsequent levels of autophagy and apoptosis [73]. Moreover, the target genes of p53 including DRAM, ISG20L1, or AEN are all reported to have the ability to regulate autophagy [74,75,76]. 

### 1.3. Autophagy and Multidrug Resistance (MDR)

The recurrence of tumors after treatment continues to represent a critical problem for clinicians. This is often due, in large part, to the multidrug resistance (MDR) response of tumor cells to chemotherapeutic agents. Therefore, there is an urgent need to develop agents with high activity against MDR, but with limited overall toxicity. The phenomena and mechanisms of MDR are summarized in great detail in many reviews [77,78,79], but in this review, we focused on how autophagy mediates MDR. Taken together with our discussion above, when autophagy exhibits cytoprotective functions, the administration of autophagy inhibitors can enhance chemotherapeutic drug sensitivity. In a Ras-NIH 3T3-Mdr cell model overexpressing p-glycoproteins (p-gp), a deficiency of autophagy was also found to facilitate necrosis and apoptosis induced by gossypol, a BH3-mimetic small molecule isolated from cottonseed, suggesting that autophagy may exhibit protective effects in drug-resistant cells [80]. Further studies have demonstrated that autophagy may be associated with resistance to a variety of anti-breast cancer drugs, such as tamoxifen, Herceptin (trastuzumab), paclitaxel (PTX), and epirubicin (EPI) [81]. These results were also validated in multidrug-resistant v-Ha-ras-transformed NIH 3T3 cell studies showing that knockout of the autophagy regulatory gene, *ATG5*, increased PTX sensitivity [82]. 

Conversely, when autophagy expresses a cytotoxic function, this could be exploited to induce MDR cancer cell death [83]. Sirichanchuen et al. found that co-treatment with cisplatin and autophagy inducer, trifluorperazine, could resensitize H460/cis cells to cisplatin-induced cell death [84]. This study is not unique, as Meschini et al. reported that vocamine, a bisindolic alkaloid from *Peschiera fuchsiaefolia*, could be utilized to overcome the resistance to doxorubicin in osteosarcoma cells by competitively inhibiting p-gp/ABCB1 and inducing autophagic cell death [85]. 

The mTOR pathway is a negative regulator of autophagy [86]. Rapamycin, an inhibitor of mTOR, activates autophagy and induces autophagic death of MDR v-Ha-ras-transformed NIH3T3 cells, drug-resistant LoVo/ADR colon cancer cells, and cisplatin-resistant cervical cancer cells [87,88,89]. GOF properties of p53 may also be involved in this process. The mutant p53 protein inhibits the generation of cytotoxic autophagy by stimulating the mTOR pathway, thereby increasing the proliferation of tumor cells [90]. In addition, when MDR cells lack the capacity to undergo apoptosis or exhibit apoptosis resistance, the agents that induce cytotoxic autophagy can also treat cancer and impede multidrug resistance through the induction of autophagy. Saikosaponin-d and Hernandezine are small molecular compounds extracted from natural plants, which promote the death of apoptosis-defective or apoptosis-resistant mouse embryonic fibroblast cells through cytotoxic autophagy [91,92]. 

Additional evidence linking autophagy to MDR suggests that lysosomal activity plays a role in tumor drug resistance. Lysosomes are at the center of cell degradative processes, responsible for decomposing proteins, polysaccharides, and lipids into their own basic structural forms [93]. Lysosomes receive extracellular or cell surface materials by endocytosis and intracellular components by autophagy. The unique acidic condition of lysosomes provides the optimal environment for the hydrolases in lysosome, and is also a precondition for the fusion of autophagosomes with lysosomes, forming the autophagolysosome and the completion of autophagic flux (i.e., degradation of the autolysosomal cargo) [94]. In addition to the widely accepted passive sequestration of hydrophobic weak base chemotherapeutics [95,96,97], other lysosome-mediated resistance mechanisms have been reported, such as the active lysosomal drug isolation mediated by the ATP-driven transporter of the ATP-binding cassette (ABC) superfamily [98,99] and the role of the lysosomal copper transporter in tumor resistance to platinum drugs [5,100,101]. Some genes regulated by p53 encode lysosomal proteins. In p53-deleted or mutant tumor cells, although autolysosome formation is not affected, a deficiency at the lysosome-mediated degradation of autophagosome cargo ensues [102]. Therefore, resistance induced by p53-dependent protective autophagy may be associated with the induction of high levels of lysosomes.

**Figure 1:**
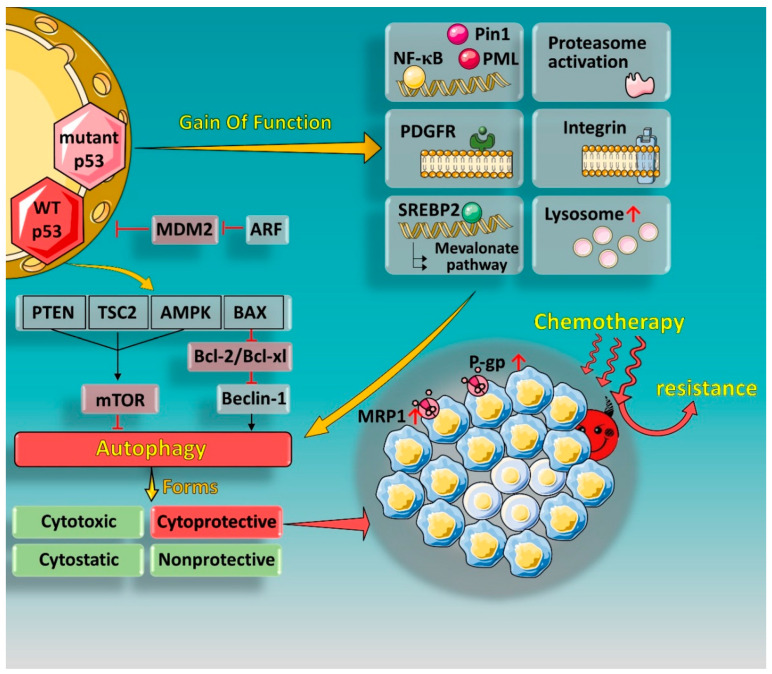
Gain of function (GOF) effect of mutant p53 and regulation of autophagy by p53 in nucleus. Certain p53 mutations (such as R248Q, R273H, R175H, and R249S) have shown GOF can further promote cancer malignance and chemoresistance. p53 mutants are able to actuate various survival signaling cascades, such as the NF-κB, PDGFRβ, mevalonate, proteasomal, or integrin pathways, and activate an independent set of target genes in cooperation with other transcription factors or cofactors (such as Pin1 and PML proteins). Nuclear p53 localization can promote autophagy by regulating the mTOR pathway in a transcription-dependent manner. Some p53 targeted genes, including PTEN, TSC2, and AMPKβ, have been reported to negatively regulate mTOR, thus promoting autophagy initiation. In addition, p53 can also be associated with autophagy through the ARF-signaling pathway. Regulation between p53 and the different functional forms of autophagy can contribute to chemotherapy resistance in tumor cells.

In general, both autophagy inhibitors and inducers are involved in the treatment of MDR-expressing cancer. There are many regulatory factors that affect the role of autophagy. Understanding the specific role of autophagy and taking advantage of autophagic functionality could potentially be an effective strategy to overcome multidrug resistance in cancer (Figure 1).

## 2. Effect of p53 Status on Autophagy and MDR

Sequencing of the cancer genome showed that 42% of the 12 tumor types analyzed carried the *TP53* mutant gene [13]. However, the mutation rate of *TP53* varies greatly among different types of tumors [9]. The importance of different types of p53 mutations leading to different therapeutic effects has been recognized; therefore, Food and Drug Adminstration (FDA)-approved drugs are now being tested in pre-clinical or clinical trials with patients stratified according to the status of p53 [10]. However, the p53 status of patients is rarely considered in clinical trials where autophagy inhibitors are used in combination. In subsequent sections, we will summarize the reported effects of p53 status on chemotherapeutic drug sensitivity and resistance in different types of tumors. 

### 2.1. Leukemia

A wide range of studies have determined that about 10% of patients with hematological malignancies have *TP53* alterations. The highest frequency was observed in acute lymphoblastic leukemia (ALL) (total: 19%; mut+del: 6%; mut only: 8%; del only: 5%) and acute myeloid leukemia (AML) (total: 13%; mut+del: 5%; mut only: 7%; del only: 1%), whereas *TP53* alterations occurred less frequently in chronic lymphocytic leukemia (CLL) (total: 8%) and myelodysplastic syndromes (MDS) (total: 7%) [103]. 

TP53-mutant AML has an extremely poor prognosis and often exhibits natural resistance to chemotherapy [104]. It was found that autophagic flux was higher in poor risk AML compared with favorable- and intermediate-risk AML, although the high autophagy flux related to *TP53* mutations, knockdown, or ectopic-overexpressing mutant p53 had no effect on autophagy flux [105]. In addition, in contrast to wtp53 AML, the autophagy inhibitor HCQ treatment did not trigger a BAX and PUMA-dependent apoptotic response in p53mut AMLs. These findings imply that the level of autophagy flux in AML might be an intrinsic property and co-treatment with autophagy inhibitors might only be effective for wtp53 AML patients [105]. 

B-cell precursor acute lymphoblastic leukemia (BCP-ALL) is the most common form of pediatric cancers. As seems to be the case with other types of leukemia, the poor prognostic group of BCR/ABL1-positive BCP-ALL appears particularly dependent on autophagy for their survival and malignant transformation. Exposure of BCP-ALL cells to irradiation triggers autophagy and cell death in a p53-dependent manner [106]. However, the combination with autophagy inhibitors, for which this situation warrants, seems to be related to the mechanism of drug action. Cheong et al. reported that autophagy inhibitors significantly increased sensitivity of the cytarabine arabinoside-resistant U937 cells, which lack the function of p53 [107,108]. Similarly, sorafenib rarely induces autophagy in wtp53 AML cells (OCI-AML3) and p53 null AML cells (HL-60), but induces protective autophagy in p53 null cells (HL-60) [109]. 

The situations in chronic leukemia are more complicated. Carew and colleges demonstrated that autophagy inhibitors augment the anti-CMLs’ activity of the histone deacetylase inhibitor suberoylanilide hydroxamic acid (SAHA) to overcome Bcr-Abl-mediated drug resistance, regardless of p53 status [110]. In the case of another drug, the Src-family protein-tyrosine kinase inhibitor, dasatinib, wtp53 CLL cells are resistant because dasatinib induces cytoprotective autophagy. In contrast, p53 mutant CLL lymphocytes are hypersensitive to dasatinib due to the low level of autophagy [111]. 

### 2.2. Gastric Cancer

*Helicobacter pylori* (*HP*) is responsible for about 90% of gastric cancer (GC) cases worldwide [112]. Recent work demonstrated that *HP* promotes p53 proteasomal degradation and inhibits USF1 expression. The low level of USF1 further drives p53 degradation and then accelerates the progression of gastric carcinogenesis, which is related to the low overall survival in GC patients [113]. This reflects the importance of activating p53 in the treatment of gastric cancer. Some p53-regulated proteins also play important roles in regulating autophagy, such as Kallikrein-related peptidase 6 (KLK6), which is p53-dependent and autophagy-related in the tumor microenvironment. Studies have shown that Auranofin, an inhibitor of thioredoxin reductase, induced resistance in gastric cancer cells may be due to overexpression of KLK6, which is affected by p53 upregulation, resulting in protective autophagy [114]. The cdk4/6 inhibitor, Palbociclib, induced p53-dependent autophagy in gastric cancer cells, and the knockdown of p53 was accompanied by a deficiency of the lysosome-mediated degradation of autophagosome cargo, resulting in autophagic blockade [102]. However, the status of p53 does not seem to play a decisive role in some anti-gastric cancer drugs. Tenovin-6 is a potent activator of p53; interestingly, the sensitivity of Tenovin-6 to gastric cancer cell lines and the initiation of autophagy were not correlated with *TP53* gene status. Meanwhile, CQ increased Tenovin-6-induced cell death also in a p53-independent manner [115].

### 2.3. Pancreatic Cancer

Pancreatic cancer is one of the most lethal types of cancer. Most patients with pancreatic cancer have genetic alterations [116], including *KRAS* [117], *TP53* [118], *CDKN2A* [119], *SMAD4* [120], *BRCA1*, and *BRCA2* [121]. Disruptions of *KRAS* and *TP53* are almost universal, with frequencies of about 70–95% [122,123] and 20–76% [124,125], respectively. Autophagy is commonly reported as a protective response for pancreatic cancer cell proliferation in vitro [126,127]. Therefore, CQ or HCQ as a chemical sensitizer (for example, against trametinib, gemcitabine, or nab-paclitaxel) is currently being actively tested in clinical trials (clinical trial: NCT01128296, NCT01506973, NCT01978184). However, these sensitizing effects could not be well reproduced in clinical trials. First, a phase II clinical trial report 5 years ago indicated that in patients with previously treated metastatic pancreatic cancer, HCQ monotherapy demonstrated negligible therapeutic efficacy [128]. Second, a recent phase II clinical trial showed that HCQ did not improve the primary end point of 12-month overall survival in patients with pancreatic cancer treated with first-line drugs, namely gemcitabine hydrochloride and nab-paclitaxel (GA) [129] (NCT01506973). In 2013, Rosenfeldt et al. showed that the status of p53 determines the development of humanized genetically modified mouse models of pancreatic ductal adenocarcinoma. They proved that treatment of mice with the autophagy inhibitor HCQ significantly accelerates tumor formation in mice containing oncogenic Kras but lacking p53 [130]. We questioned whether these unsatisfactory clinical trials could be related to p53 mutations; however, this does not seem to be the case. Yang et al. demonstrated that CQ inhibited the proliferation of pancreatic cancer transplanted tumors, independently of p53 status [131]. Analysis of patients who had undergone combined treatment of HCQ and GA showed that there was no significant correlation between the prognosis of patients after treatment and *TP53* mutational status [129]. This suggests that both autophagy inhibitors alone and in combination often produce disappointing results in the clinic, which may not be related to the status of p53. Nevertheless, there have been a few promising outcomes such as a recent study showing that ERK inhibition may enhance the dependence of pancreatic ductal cancer on autophagy [132]. Therefore, blocking the ERK pathway and combining autophagy inhibitors into clinical practice could prove to be an effective strategy in the treatment of pancreatic ductal carcinoma.

### 2.4. Colorectal Cancer

Colorectal cancer is the third most common cancer in the world and a leading cause of cancer-related deaths [133]. Comprehensive genomic analysis has revealed that p53 mutations exist in about 60% of colorectal cancers, with most being of the missense-type at “hot spots”, which indicates that the mutated p53 has carcinogenic effect through the GOF mechanism [134]. However, the effect of p53 status on chemosensitivity is not consistent. Early studies by Violette et al. found that the resistance of 5-fluorouracil to 8 different kinds of colon cancer cells was related to the relative levels of BCL-2, BCL-x(L), and BAX, but not to the status of p53 [135]. However, clinical studies have shown that colorectal tumors with mutant p53 have a weak or absent response to 5-fluorouracil therapy. Patients with wtp53 colorectal tumors have a longer survival period than those with mutant p53 tumors [136]. This suggests that when chemotherapeutic drugs act on the whole body, p53 may be associated with many other factors, such as tumor microenvironment or autophagy, and the “gatekeeper” role of p53 may be more obvious. 

The relationship between p53 status and autophagy in colon cancer has also been studied by the Kroemer laboratory. These investigators reported that knock out p53 in HCT-116 cells improved mouse survival by inducing rather than blocking autophagy [17], and that re-transfection with wtp53 inhibited baseline autophagy [137]. Since these reports, more studies have explored the relationship between the activity of anti-colon cancer agents and the regulation of p53 and autophagy. Compared with HCT-116 p53+/+ cells, HCT-116 p53−/− cells are more sensitive to Crocin (the bioactive molecule of saffron), which is associated with its induction of defective autophagosome formation in HCT-116 p53−/− cells [138]. Betulinic acid (BA), a naturally occurring pentacyclic triterpene, has demonstrated antitumor properties in several human cancers [139]. BA interferes with the induction of protective autophagy by degrading mutant p53 through a ubiquitin-mediated degradation pathway, thus inducing apoptosis and promoting the death of colon cancer cells [140]. The protective autophagy in colon cancer correlated with p53 status may be associated with the loss of the ribosomal protein uL3. Ribosomal protein uL3 has been shown to be a key sensor of nucleolar stress induced by a variety of chemotherapeutic drugs (such as 5-fluorouracil, oxaliplatin, and actinomycin D (Act D)) in p53-deficient colon cancer cells [141,142,143]. Pecoraro et al. further demonstrated that loss of uL3 activated cytoprotective autophagy and in turn mediated resistance to Act D in colon cancer [144]. In addition, Zhang et al. recently proposed that p53 can regulate Ten-eleven-translocation 2 (TET2), a protein that regulates DNA damage by maintaining the DNA repair pathway, through the autophagic degradation pathway. Studies have shown that knockout of TET2 in p53 null colon cancer cells can reverse resistance to chemotherapeutic drugs such as doxorubicin and cisplatin. This provides a potential explanation for drug resistance mechanisms in p53 null colon cancer cells, specifically that loss of p53 leads to lowered degradation of TET2 protein in the cytoplasm, but more accumulation in the nucleus during doxorubicin or cisplatin treatment. Nuclear TET2 protects the genome from DNA damage caused by doxorubicin or cisplatin; ultimately, promoting the growth and survival of colon cancer cells and contributing to chemoresistance [145].

### 2.5. Liver Cancer

Hepatocellular carcinoma (HCC) is the most common form of liver cancer in adults, accounting for ~85–90% of liver cancer patients [146]. Major risk factors include chronic infections with hepatitis B (HBV) or C (HCV) virus, dietary aflatoxin B_1_ (AFB_1_) toxins, or alcohol consumption [147,148]. *TP53* mutations are exhibited in ~25–30% of HCC patients [149], more than ~50% in AFB_1_-related HCC patients, and ~45% of HBV-related HCC patients [150]; thus, detection of point mutations in TP53 is considered a biomarker for AFB_1_ exposure and risk for HCC. Transversion of G:C to T:A at the third position of codon 249^ser^ was detected in the serum DNA of HCC patient biopsies in areas of high AFB_1_ exposure and HBV endemic areas [151,152]. This *TP53 249^ser^* mutant was shown to inhibit wt p53-mediated apoptosis and facilitate tumor cell growth when transfected into p53 null liver cancer cells [153,154]. 

HBV is a DNA virus that infects hepatocytes, causing liver injury and hepatocyte cell death, and which can promote tumorigenesis. Its DNA codes for four distinct proteins, envelope protein, nucleocapsid (core) protein, viral reverse transcriptase, and the X gene of HBV (HBx) protein [147]. HBx binds to p53 and decreases p53 binding to XPB [155,156], which is important for nucleotide excision repair interaction between HBx and p53. Furthermore, HBx also inactivates p53-dependent activity, such as p53 mediated transcription of cell cycle regulators, repression of *TP53* transcription and p53-activated apoptosis [154,157,158,159,160]. HBx binding to PI3KC3 has also been shown to enhance viral replication by inducing autophagy in hepatoma cells transfected with HBV [161]; furthermore, Mizui et al. demonstrated that autophagy inhibition can limit HCV replication [162]. Furthermore, GOF p53 mutations were demonstrated to alter apoptosis induction in hepatocellular carcinoma. Hep3B cells (p53 null) transfected with varying GOF p53 mutations and p53wt plasmids exhibited an anti-apoptotic gain of function. Mutant p53 was able to repress *CD95* (fas/APO-1) gene transcription, as well as repress BAX expression, thus attenuating mitochondrial mediated apoptotic pathways and extrinsic apoptotic pathways [163].

Excessive inflammation and tissue damage due to chronic infection by HBV or HCV or alcohol consumption are major contributors to hepatocarcinogenesis. Autophagy induction can contribute to alleviation of some of this toxic stress by clearing damaged protein accumulation, dysfunctional mitochondria and genomic stress [164]. TAK1-mediated activation of autophagy prevented excessive lipid accumulation, while *Tak1*-depletion resulted in lipid accumulation, hepatosteatosis, and tumorigenesis [165]. In contrast, high basal autophagy can also promote tumorigenesis and chemoresistance [166,167]. Du et al. demonstrated that autophagy inhibition through ATG7 silencing and CQ pretreatment sensitized HepG2 hepatocarcinoma cells to oxaliplatin treatment and enhanced apoptotic cell death [168]. 

Although the indicated studies suggest that autophagy is cytoprotective in liver cancer, autophagy has also been shown to play dual functional roles in this disease. Studies by Zhang et al. showed that resveratrol inhibited proliferation and migration of HCC cells through the promotion of autophagy. Furthermore, the anti-tumor effects of resveratrol were attributed to autophagy induction through resveratrol-mediated p53 activation, as well as inhibition of PI3K/AKT [169]. Treatment with 3-MA, an autophagy inhibitor, negated resveratol cytotoxic effects on HCC cell proliferation, invasion and migration. Similarly, Wang et al. demonstrated in HCC cell lines that exposure to fangchinoline, a bisbenzylisoquinoline alkaloid shown to induce cell cycle arrest in breast and prostate cancer cell lines [170], induced autophagy in a p53/sestrin2/AMPK-dependent manner and induced autophagic cell death in HepG2 and PLC/PRF/5 cell lines [171]. Consequently, it is feasible that targeting autophagy may potentiate chemosensitization and induce cell death in hepatocarcinoma cells [172,173].

### 2.6. Lung Cancer

Lung cancer is the second most common type of cancer and is responsible for the most cancer-related deaths [174]. There are two major types of lung cancers: Non-small cell lung cancer (NSCLC), which accounts for ~80–85% of new cases, and small cell lung cancer (SCLC), which contributes to ~10–15% of lung cancer cases [175,176]. Lung cancers have a high p53 mutation rate, of approximately 46% in lung adenocarcinoma and 81% in squamous cell carcinoma. Furthermore, lung cancers also demonstrate a higher percentage of mutations within specific amino acid residue extensions (157, 175, 248, and 273), which is further exacerbated by smoking [177]. The most common mutations in lung cancer include KRAS and p53 mutations; moreover, tumors with p53 mutations generally have poor prognosis and chemoresistance [178,179]. Mutant p53 was shown to upregulate Nrf2 activity, a transcription factor that codes for antioxidant proteins, multidrug resistance, and other proteins that are induced to protect against oxidative and chemotoxic damage [180]. In this study, Tung et al. demonstrated that wtp53 suppressed Nrf2 promoter activity in NSCLC cells; however, in p53 mutant cells promoter activity was not suppressed, resulting in increased mRNA levels of Nrf2 and the anti-apoptotic proteins, Bcl-2 and Bcl-xL, ultimately contributing to cisplatin resistance [180].

p53-regulated processes, such as autophagy, have also been shown to mediate chemoresistance in lung cancer [181,182]. Studies by Saini et al. examined the effects of p53 GOF mutants on cancer cell resistance to chemotherapy and proteasomal inhibition utilizing H1299 p53 null NSCLC cells transfected with either wildtype p53, R273H mutant GOF, or empty vectors [183]. They demonstrated that R273H-p53 mutant cells were significantly less sensitive to cisplatin and 5-FU, as well as conferring resistance to these therapies. Dual treatment with a proteasomal inhibitor, peptide aldehyde N-acetyl-leu-leu-norleucinal (ALLN), and autophagy inhibition did not sufficiently promote cell death in R273H-p53 mutant cells; however, activation of autophagy by serum starvation or rapamycin exposure enhanced cell killing through increased autophagosome accumulation and ROS levels. Wu et al. demonstrated that cisplatin-refractory A549 lung adenocarcinoma cells exhibited greater basal autophagy compared to parental A549 cells; furthermore, treatment with cisplatin increased autophagy induction [184]. Autophagy inhibition with CQ in cisplatin-resistant A549 cells induced apoptosis and resensitized these cells to cisplatin. These data indicate that autophagy may play a role in the development and maintenance of chemoresistance in lung cancer. However, in a recent paper, our laboratory demonstrated differential autophagic function induced in H460 p53 wt and H460crp53 (CRSPR/Cas 9 knockout of p53) in response to cisplatin [185]. Specifically, we showed that H460p53 wt cells were significantly more sensitive than p53 knockout cells; furthermore, this difference in sensitivity was attributed to autophagic function. H460p53 wt cells exhibited nonprotective autophagy in response to cisplatin treatment, whereas H460crp53 cells demonstrated cytoprotective autophagy. Furthermore, inhibition of autophagy was sufficient to sensitize H460crp53 cells and induce apoptotic cell death to the same extent as occurred in its p53 wt counterpart exposed to cisplatin in the absence of autophagy inhibition [185]. These studies highlighted the complexity of p53 function and its relationship to autophagic function and chemotherapy resistance. 

### 2.7. Breast Cancer

Breast cancer is the most common cancer amongst the female population and is the second leading cause of cancer-associated deaths amongst women [174]. There are a number of various breast cancer subtypes and *TP53* status can vary amongst these subtypes. *TP53* mutations can be found in 26% of luminal tumors (17% of luminal A and 41% of luminal B, 69% of molecular apocrine tumors, 88% of basal-like carcinomas, and roughly 50% of HER2 amplified tumors) [186]. Schimmelpenning et al. demonstrated overexpression of mutant p53 in highly proliferative human mammary adenocarcinomas that were more aggressive compared to their p53 negative counterparts [187].

Cui et al. demonstrated that p53/DRAM signaling contributed to radiation-induced autophagic cell death and apoptosis in MCF7 breast cancer cells [74,188]. Alternatively, Cordani et al. showed that a GOF p53 mutant inhibited autophagic vesicle formation through APMK inhibition and mTOR stimulation, contributing to reduced apoptosis and prolonged survival [90]. The authors showed that patients with p53 mutations with low autophagic gene signatures had significantly poorer prognoses compared to their counterpart with higher autophagic gene signatures with p53 mutations. 

Almost 70% of breast cancer patients have estrogen receptor-positive (ER+) breast cancer [189]. While selective estrogen receptor modulators, such as tamoxifen, aromatase inhibitors, and fulvestrant or falsodex (ICI), are effective in most patients, resistance to these therapies still develops, leading to disease recurrence. Mechanisms for acquired resistance to endocrine therapy in ER+ breast cancer remain poorly understood; however, pre-clinical data suggests that autophagy may be a contributing factor [190]. Studies by Sun et al. showed that acquired multidrug resistance to simultaneous paclitaxel and vinorelbine exposure in MCF-7 and SK-BR-3 is facilitated by autophagy through apoptosis inhibition [81]. Additionally, Cook et al. utilized tamoxifen (TAM)-resistant MCF-7 cells and faslodex-resistant/tamoxifen cross-resistant LCC9 ER+ breast cancer cells to examine the effects of autophagy inhibition on anti-estrogen therapy [191]. Treatment with CQ restored sensitivity to TAM in resistant tumors, both in vivo and in vitro; however, these effects were not paralleled with the combination treatment of CQ + ICI in vivo. TAM and ICI treatment in combination with CQ was sufficient to reduce cell viability in vitro in both cell lines, suggesting that autophagy plays a cell-autonomous role in anti-estrogen therapy resistance. However, the reduced in vivo efficacy of ICI + CQ was attributed to cell-mediated immunity, specifically to lowered peripheral macrophage infiltration, suggesting that cell-nonautonomous roles of autophagy must also be considered. Chollat–Namy et al. showed reactivation of wtp53 function in p53-mutated tumors using CP-31398, a small molecule that stabilizes p53 conformation and promotes autophagy induction via transcriptional activation of sestrin-AMPK and mTOR inhibition. Furthermore, reactivation enhanced cytotoxic killing by cytotoxic T lymphocytes (CTL) and NK cells and facilitated granzyme-B-mediated mitochondrial permeabilization and caspase activation through the sequestration of anti-apoptotic proteins in autophagosomes [192]. The authors demonstrated p53 reactivation induced autophagy through the sestrin/AMPK/mTOR axis and facilitated granzyme B and NK cell-induced mitochondrial poration and subsequent cell death through sequestration of anti-apoptotic proteins, such as Bcl-X_L_ and XIAP. These data suggest that autophagy plays a key role in the development of resistance to ER+ breast cancer and hormonal therapy, as well as mitigating immune responses in the tumor microenvironment. 

Triple negative breast cancer (TNBC) is characterized by its lack of expression of estrogen receptor, progesterone receptor, and human epidermal growth factor receptor 2, and accounts for ~10–15% of breast cancers [189]. It is noteworthy that about 80% of patients with TNBC have the p53 mutation [193,194,195]. Similar to what was mentioned earlier, p53 mutations at different sites have different effects on autophagy levels and chemosensitivity. The histone deacetylase inhibitor, SAHA, induces autophagy while degrading mutp53 in the TNBC MDA-MB-231 breast tumor cell line (mutp53-R280K), and autophagy inhibitors enhance the cytotoxic effect of SAHA. Interestingly, it was also found in this study and a study mentioned above that the same agents often have different outcomes. For p53 mutant colon cancer cell DLD1 (mutp53-S241F), SAHA only degraded mutp53 but did not induce high levels of autophagy; therefore, autophagy inhibitors had no significant effect on the activity of SAHA [196]. For CML cells, p53 knockdown showed no effect on the sensitivity to SAHA and autophagy inhibitors’ combination treatment [110]. 

Chittaranjan et al. examined the role of autophagy in TNBC resistance to chemotherapy [197]. Due to the lack of viable receptor expression, targeted therapy options are limited for these patients, and while patients initially respond to chemotherapy, resistance can develop, resulting in poor outcomes and aggressive disease. Utilizing TNBC cell lines both sensitive and resistant to epirubicin and anthracycline derivatives, they assessed basal autophagy and the effects of autophagy inhibition in both parental and resistant cell lines. Their data showed greater basal autophagy in MDA-MB-231-R8- and SUM159PT-RRR75-resistant cell lines compared to the parental anthracycline-sensitive lines. Furthermore, pharmacological (HCQ and CQ) and genetic inhibition (shATG5/7) significantly sensitized both anthracycline-sensitive and anthracycline-resistant cell lines, suggesting that autophagy is cytoprotective in multidrug resistance to anthracyclines in TNBC. Collectively, these data suggest that p53 and autophagy play complex roles in breast cancer, both in a cell autonomous, as well as cell non-autonomous, manner (Table 1).

## 3. Conclusions and Future Directions

The role of p53 in tumor resistance has long been recognized and extensively studied as an anti-tumor target. p53 functions in modulating various cellular fates and responses to chemotherapy and radiation, including apoptosis, autophagy, and senescence. However, p53 status is often neglected in the clinical therapeutic regimens prescribed for the treatment of patient tumors. From the discussion above, we may safely draw the conclusion that a number of factors may contribute to the clinical outcome of chemotherapeutics and patient tumor responses to treatment. The heterogeneity in patient responses to therapy may depend on p53 expression, the type of mutation, the site of the mutation, the type of tumor, and the chemotherapeutic utilized. These various factors may partly explain the inconsistency of clinical responses to autophagy inhibitors. Further preclinical studies are warranted to consider these factors to address the efficacy of the use of autophagy inhibitors as a means to sensitize tumor cells to chemotherapy. 

Given the multifunctional nature of autophagy, it is essential to further interrogate the functional form of autophagy induced in response to chemotherapy and radiation within patients, as well as to identify cellular mechanisms involved in determining autophagic function. While p53 status can modulate autophagy, how it mechanistically contributes to the “decision” of the functional form of autophagy induced in response to a specific therapy has yet to be determined. Preclinical studies delving deeper into the contributions of p53 status on autophagic function will provide valuable insights and may lead to the identification of novel therapeutics and biomarkers, allowing for the selection of patient tumors responsive to chemosensitization through autophagy inhibition. Individualized treatment of patients with the incorporation of p53 status and optimal therapeutic treatment windows are necessary considerations for future clinical trials utilizing autophagy inhibitors. 

## Figures and Tables

**Table 1:** This table demonstrates the relationship between p53 status and autophagy and its effects on chemosensitivity.

Tumor	Models/Cells	Drug/Agent	Sensitivity	Autophagy Level	The Sensitivity after Use Autophagy Inhibitor	Autophagy & p53	Reference
AMLs	Patients; HL60, K562, THP1, OCIM3, MOLM13, and NB4	HCQ	mutp53 < wtp53	mutp53 > wtp53 (but the status of p53 have no effect on autophagy flux)	N/A	TP53mut AML cells show decreased sensitivity for short-term treatment with HCQ and an impaired upregulation of the apoptotic genes PUMA and BAX, indicating that the initial apoptotic response in these cells is strongly impaired	[105]
	Primary acute myeloid leukemia blasts and OCI-AML3, MOLM, MV4-11, HL60, or NB4	Sorafenib	p53-independent	N/A	Cells lack of p53 function, increased sensitivity (online Supplementary Figure S5)	N/A	[109]
BCP-ALL	cytarabine-resistant U937 leukemia cells	Cytarabine	N/A	N/A	Cells lack of p53 function, increased sensitivity	N/A	[106]
CML	Ba/F3 p210 and Ba/F3 T315I cells	suberoylanilide hydroxamic acid (SAHA)	p53-independent	N/A	Increased sensitivity, independent of p53	N/A	[110]
CLL	Patients	Dasatinib	mutp53 > wtp53	mutp53 < wtp53	mutp53 (non-protect); wtp53 (CQ increase sensitivity; 3-MA or pifithrin same trend increase or no change)	mutp53 CLL lymphocytes are hypersensitive to dasatinib because of the lack of dasatinib-induced p53 dependent autophagy where mutated p53 exerts an inhibitory effect on dasatinib-induced p53-independent autophagy (wtp53 induces autophagy, mutp53 low autophagy)	[111]
Gastric cancer	AGS	Palbociclib	p53-independent	p53-independent	p53-independent	N/A	[115]
Pancreatic cancer	Patients	Gemcitabine and nab-Paclitaxel	p53-independent	NA	With HCQ, p53-independent	N/A	[129]
Colon cancer	SW620 Ad300 cells and SW620 cells; p53+/+ and p53−/− HCT116 cells	Cryptotanshinone (CTS) and dihydrotanshinone (DTS)	Null p53 = wtp53	Resistance cell > SW620 cells (SW620 cells apoptosis > resistance cell)	Null p53 = wtp53, no change	CTS and DTS induced p53-independent apoptosis and autophagy in colon cancer cells	[198]
	HCT-116 cell	Crocin (the bioactive molecule of saffron)	Null 53 > wtp53	N/A	Baf A1 increased the sensitivity of p53 wt HCT-116 cells, no change in p53 null HCT-116 cells	N/A	[138]
	HCT-116 cell	Betulinic acid (BA), a naturally occurring pentacyclic triterpene	mutp53 expression enhanced HCT-116 resistance to BA	N/A	CQ and ATG5 siRNA increased the BA-induced sensitivity in a p53-independent manner	N/A	[140]
Liver cancer	Huh-7 (mutp53) and SMMC-7721 (wtp53)	Oxaliplatin	wtp53 < mutp53	wtp53 = mutp53	Exposure to CQ or 3-MA significantly increased oxaliplatin-induced cell death in both wtp53 and mutp53 cells; genetic autophagy inhibition also concurred with this increase in cell death in both cell lines when autophagy is knocked down	Oxaliplatin induced p53-independent autophagy in HCC	[199]
	HepG2 (wtp53) and Huh-7 (mu p53)	Sorafenib	p53-independent	N/A	Autophagy inhibition (BafA1 and 3-MA) reduced sensitivity in sorafenib-resistant HepG2 and Huh-7 cells when compared to parental cells;	N/A	[200]
Lung cancer	H1299 (p53 null cells) transfected with wtp53 or R273H GOF p53	5-FU and cisplatin; most studies performed with proteasomal inhibitor, peptide aldehyde N-acetyl-leu-leu-norleucinal (ALLN)	R273H GOF p53 > wtp53	inhibition of R273H GOF p53 increased autophagy induction	treatment with rapamycin (mTOR inhibitor) or serum starvation (autophagy-inducers) enhanced ALLN-induced cytotoxicity in R273H GOF p53 H1299 cells; furthermore, inhibition of autophagy with CQ did not significantly alter ALLN-induced cell death in R273H GOF p53 H1299 cells	Enhancing autophagy can R273H GOF p53 cells sensitize to ALLN treatment by promoting ROS and ERK signaling	[183]
Breast cancer	MDA-MB-231(mutp53-R280K) and DLD1 (mutp53-S241F)	Histone DeACetylases inhibitor, suberoylanilide hydroxamic acid (SAHA)	N/A	MDA-MB-231(mutp53-R280K) > DLD1 (mutp53-S241F)	Autophagy inhibition (BafA1) enhanced cytotoxicity of SAHA in MDA-MB-231 cells	SAHA induced autophagy induction, which promoted degradation of mutp53 in MDA-MB-231 cells but not in DLD1 cells; autophagy inhibition stabilized mutp53 in MDA-MB-231 cells	[196]
	MDA-MB-231 (mutp53-R280K) and SUM159PT	Epirubicin	N/A	anthracycline-resistant > anthracycline-sensitive cell lines	Pharmacological (CQ or BafA1) and genetic inhibition (siATG5 or siATG7) significantly sensitized both anthracycline-sensitive and anthracycline-resistant cell lines; cytoprotective autophagy induced	N/A	[197]

N/A, not applicable.

## Abbreviations

GOF	Gain of function
MDR	Multidrug resistance
TIS	Therapy-induced senescence
MDR1	Multidrug resistance gene 1
wt	Wildtype
NSCLC	Non-small cell lung cancer
CQ	Chloroquine
HCQ	Hydroxychloroquine
ATG	Autophagy-related genes
MOMP	Mitochondrial outer membrane permeabilization
HMGB1	High mobility group box 1
PTX	Paclitaxel
EPI	Epirubicin
ABC	ATP-binding cassette
ALL	Acute lymphoblastic leukemia
AML	Acute myeloid leukemia
CLL	Chronic lymphocytic leukemia
MDS	Myelodysplastic syndromes
BCP	B-cell precursor acute lymphoblastic leukemia
SAHA	Suberoylanilide hydroxamic acid
HP	Helicobacter pylori
GC	Gastric cancer
KLK6	Kallikrein-related peptidase 6
GA	Gemcitabine hydrochloride and nab-paclitaxel
5-FU	5-fluorouracil
BA	Betulinic acid
Act D	Actinomycin D
TET2	Ten-eleven-translocation 2
HCC	Hepatocellular carcinoma
HBV	Hepatitis B
HCV	Hepatitis C
AFB_1_	Aflatoxin
HBx	X gene of HBV
3-MA	3-methyladenine
SCLC	Small cell lung cancer
ALLN	Peptide aldehyde N-acetyl-leu-leu-norleucinal
ICI	Fulvestrant or falsodex
TAM	Tamoxifen
CTL	Cytotoxic T lymphocytes
TNBC	Triple negative breast cancer
